# Incidence of skin cancer development in pancreatic islet cell transplant patients: A retrospective study

**DOI:** 10.1016/j.jdin.2021.01.004

**Published:** 2021-04-01

**Authors:** Iviensan F. Manalo, Valerie Truong, Taryn DeGrazia, Alexander L. Kollhoff, Hayley Braun, Howa Yeung, Travis W. Blalock

**Affiliations:** a Department of Dermatology, Emory University School of Medicine, Atlanta, Georgia; b Augusta University/University of Georgia Medical Partnership, Athens, Georgia; c Emory University School of Medicine, Atlanta, Georgia; d Division of Dermatology, Atlanta VA Medical Center, Decatur, Georgia; e Winship Cancer Institute, Emory University, Atlanta, Georgia

*To the Editor:* Long-term survivors of solid organ transplants are at an increased risk of malignancies, most commonly skin cancer.^1^ While the risks of heart, lung, kidney, kidney-pancreas, liver, and hematopoietic cell transplant recipients are established, there are limited data on pancreatic islet cell transplant (ICT) recipients.^2^ ICT is an effective beta-cell replacement therapy performed to normalize glycemic control in patients with type 1 diabetes mellitus^3^ and/or as a treatment for patients with chronic pancreatitis.^4^ This study aims to identify and compare the incidence of nonmelanoma skin cancer (NMSC) in allogeneic and autologous ICT recipients.

This retrospective study included pancreatic ICT recipients examined at Emory University Hospital from between January 1, 2003 to December 31, 2010. The patients were identified from the hospital's database and the Organ Procurement Transplant Network. The exclusion criteria included known genetic diseases conferring predisposition to skin cancer, immunosuppression due to conditions other than the use of antirejection medications, transplant failure with immunosuppressive medication cessation, and death without any dermatologic medical record. Between 2019 and 2020, research personnel performed chart reviews of the eligible patients, specifically, their demographics and transplant history. Skin cancer histories were obtained through telephonic surveys or secure patient portal messages. In an effort to minimize survey response bias, the patients' dermatologic chart records were independently reviewed. Data were summarized as means with standard deviations or as percentages. The Fisher exact and independent 2-tailed *t* tests, with *P* values < .05 considered statistically significant, were performed to compare allogeneic and autologous groups. Poisson modeling was used to compare incidence rates. Cumulative incidence curves of time for the first NMSC occurrence were plotted.

Of the total patients eligible (21 allogeneic and 13 autologous), information was collected from 6 patients in the allogeneic and 6 in the autologous group. The demographics and case information detailed in Table I demonstrated no significant difference in sex, race, or age during first ICT. Although not significant, overall, skin cancer developed in a higher proportion of patients in the allogeneic group than in patients in the autologous group (5 vs 2, or 83.3% vs 33.3%, respectively). The average age at the first NMSC occurrence was significantly lower in the allogeneic group (55.8 years vs 70 years, respectively; *P* < .05). The first NMSC in the allogeneic group occurred 2 years after transplant compared to 10 years in the autologous group. The strength and dose of immunosuppression with mycophenolate mofetil or tacrolimus did not appear to correlate with how soon NMSC developed in patients undergoing transplant. Although not significant, a large effect size separating the cumulative incidence curves of time for the first NMSC occurrence between the 2 groups was observed. Additionally, although 30% of the patients in the autologous group were nonwhite, a sensitivity analysis excluding those patients did not change the cumulative incidence curve (Fig 1).

**Table I:** Demographic characteristics of pancreas islet cell transplant recipients. Despite similar demographic profiles and the fact that NMSC incidence rates between the 2 groups were nonsignificant, the average age at the first NMSC occurrence was significantly lower in the allogeneic group

	Allogeneic transplant recipients, n	Autologous transplant recipients, n	*P* value
Total number of participants	6	6	
Sex (%)			
Male	2 (33.3)	1 (16.7)	1.00
Female	4 (66.7)	5 (83.3)	
Age at first transplantation (years)			
Mean (SD)	49.8 (5.8)	47.5 (10.6)	.64
Median (range)	48.5 (45-60)	47.5 (33-61)	
Race			
White (%)	6 (100)	4 (66.7)	.45
Nonwhite (%)	0	2 (33.3)	
History of nonmelanoma skin cancer prior to transplant (%)	0	0	
Melanoma developed after first islet cell transplant (%)	0	0	
Nonmelanoma skin cancer developed after first transplant (%)	5 (83.3)	2 (33.3)	.24
Incidences of cutaneous squamous cell carcinoma during follow-up period (n)	7	1	
Incidences of basal cell carcinoma during follow-up period (n)	1	1	
Incidence rates of NMSC	0.106	0.026	.12
Developed Merkel cell carcinoma or any other skin cancer besides melanoma and NMSC after transplant (%)	0	0	
On long-term antirejection immunosuppression (mycophenolate mofetil and tacrolimus) for >1 year (%)	6 (100)	0	
Age at the first skin cancer occurrence after transplant (years)			
Mean (SD)	55.8 (5.5)	70 (4.2)	.02
Median (range)	56.0 (47-61)	70 (67-73)	
Years to skin cancer development after transplant (years)			
Mean (SD)	8.0 (6.5)	11 (1.4)	.57
Median (range)	5 (2-15)	11 (10-12)	

*NMSC*, Nonmelanoma skin cancer; *SD*, standard deviation.

**Fig 1:**
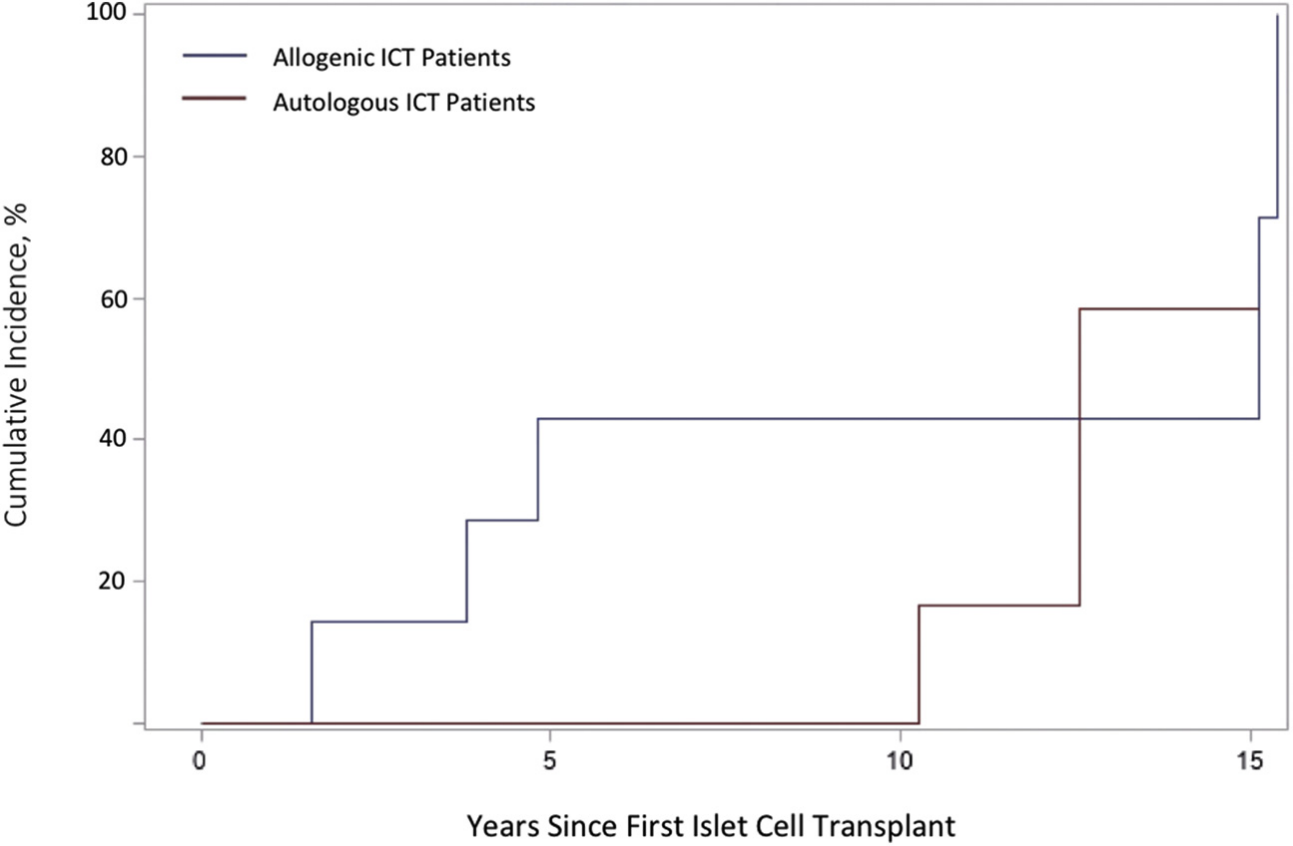
Cumulative incidence of the first NMSC occurrence after the first ICT. Although not significant, a large effect size separating the cumulative incidence curves of time for the first NMSC occurrence between the 2 groups was observed. Sensitivity analysis excluding nonwhite patients yielded the same cumulative incidence curve. *ICT*, Islet cell transplantation; *NMSC*, nonmelanoma skin cancer.

Our study reports the risk of NMSC in a pancreatic ICT population. The limitations included paucity of data because ICTs are a rare occurrence and subsequently lead to an underpowered data analysis. However, the allogeneic group had increased incidences of squamous cell carcinomas compared with the autologous group, which was in agreement with the established risk of squamous cell carcinomas in solid organ transplant recipients thought to be related to immunosuppression. The basal cell carcinoma incidence in both the groups can most likely be accounted for because it is the most common skin cancer worldwide and is not caused by immunosuppression.^1^ Despite similar demographic profiles, the average age at the first NMSC occurrence was significantly lower in the allogeneic group, suggesting that chronic immunosuppressive medications may be a risk factor for the development of NMSC in this population. Allogeneic ICT recipients likely warrant an evaluation and assessment strategy similar to that for other solid organ transplant recipients.^5^ As the indication for ICTs expands, it will become increasingly important to be aware of the risks of NMSC with long-term immunosuppression.

## Conflicts of interest

None disclosed.
